# Essential role of *Plasmodium* perforin-like protein 4 in ookinete midgut passage

**DOI:** 10.1371/journal.pone.0201651

**Published:** 2018-08-13

**Authors:** Elena Deligianni, Natalie C. Silmon de Monerri, Paul J. McMillan, Lucia Bertuccini, Fabiana Superti, Maria Manola, Lefteris Spanos, Christos Louis, Michael J. Blackman, Leann Tilley, Inga Siden-Kiamos

**Affiliations:** 1 Institute of Molecular Biology and Biotechnology, Foundation for Research and Technology—Hellas, Heraklion, Greece; 2 The Francis Crick Institute, London, United Kingdom; 3 Department of Biochemistry and Molecular Biology, The University of Melbourne, Melbourne, VIC, Australia; 4 ARC Centre of Excellence for Coherent X-ray Science, The University of Melbourne, Melbourne, VIC, Australia; 5 Bio21 Molecular Science and Biotechnology Institute, The University of Melbourne, Melbourne, VIC, Australia; 6 Biological Optical Microcopy Platform, The University of Melbourne, Melbourne, VIC, Australia; 7 National Centre for Innovative Technologies in Public Health, National Institute of Health, Rome, Italy; 8 Faculty of Infectious and Tropical Diseases, London School of Hygiene and Tropical Medicine, London, United Kingdom; Bernhard Nocht Institute for Tropical Medicine, GERMANY

## Abstract

Pore forming proteins such as those belonging to the membrane attack/perforin (MACPF) family have important functions in many organisms. Of the five MACPF proteins found in *Plasmodium* parasites, three have functions in cell passage and one in host cell egress. Here we report an analysis of the perforin-like protein 4, PPLP4, in the rodent parasite *Plasmodium berghei*. We found that the protein is expressed only in the ookinete, the invasive stage of the parasite formed in the mosquito midgut. Transcriptional analysis revealed that expression of the *pplp4* gene commences during ookinete development. The protein was detected in retorts and mature ookinetes. Using two antibodies, the protein was found localized in a dotted pattern, and 3-D SIM super-resolution microcopy revealed the protein in the periphery of the cell. Analysis of a C-terminal mCherry fusion of the protein however showed mainly cytoplasmic label. A *pplp4* null mutant formed motile ookinetes, but these were unable to invade and traverse the midgut epithelium resulting in severely impaired oocyst formation and no transmission to naïve mice. However, when *in vitro* cultured ookinetes were injected into the thorax of the mosquito, thus by-passing midgut passage, sporozoites were formed and the mutant parasites were able to infect naïve mice. Taken together, our data show that PPLP4 is required only for ookinete invasion of the mosquito midgut. Thus PPLP4 has a similar role to the previously studied PPLP3 and PPLP5, raising the question why three proteins with MACPF domains are needed for invasion by the ookinete of the mosquito midgut epithelium.

## Introduction

Perforin-like proteins are characterized by the presence of a membrane attack complex/perforin (MACPF) domain. This class of proteins are structurally related, although with little similarity in primary amino acid sequence [1]. They are found in a large number of organisms and are structurally related to the prokaryotic cholesterol-dependent cytolysins. MACPF proteins have the ability to insert into target membranes as oligomers, forming membrane-spanning pores. Pore formation is initiated by the recognition of the target membrane by the protein monomer, followed by binding to the membrane. Insertion of oligomers into the membrane results in the formation of water-filled pores which allow the passage of small molecules or proteins, depending on the context. The outcome of pore formation can be cell death, membrane rupture, or activation of cell signaling processes (for reviews see [2,3]).

In apicomplexan parasites, perforin-like proteins have been studied by reverse genetics approaches and found to have various functions. The *Toxoplasma gondii* perforin-like protein 1 (TgPLP1) was shown to have a role in egress of tachyzoites from the host cell, by destabilizing the parasitophorous vacuole membrane (PVM) surrounding mature tachyzoites [4]. A detailed functional analysis of TgPLP1 investigating the role of its three domains (the N-terminus, the central MACPF domain and the C-terminus) [5] revealed that the N-terminal part of the protein is not essential, but likely plays a role in enhancing membrane binding. The C-terminal domain, on the other hand, is critical for the function of the MACPF domain *in vivo*. The study also showed that the MACPF domain inserts into host cell membranes as a high molecular weight complex. PPLP2 in *Plasmodium berghei* and *P*. *falciparum* has an important function in egress of the gametocyte from the host cell, as a mutant lacking the protein is trapped inside the red blood cell. This suggests that PPLP2 functions specifically in destabilizing the host cell membrane [6,7]. Three PPLPs in *Plasmodium* have functions in cell passage, two in the ookinete stage (PPLP3, or Membrane Attack Ookinete Protein, and PPLP5) while the SPECT2 (Sporozoite Microneme Protein Essential for Cell Traversal or PPLP1) protein is involved in cell traversal by the sporozoite [8–12]. The function of these three proteins is consistent with the notion that they form a pore in a target membrane. The ookinete proteins PPLP3 and 5 are necessary for the invasion of the midgut epithelial cells, as determined by microscopy studies which showed that ookinetes lacking these proteins remain attached to the epithelium cells, unable to invade [8,10]. Independent analysis of *P*. *berghei*, *P*. *yoelii* and *P*. *falciparum* mutants lacking PPLP1 found that the mutants were unable to traverse cells, a necessary step before invasion of hepatocytes *in vivo* [9,11,12]. Furthermore, PPLP1 was showed to have an important function in parasite exit from the transient vacuoles that are formed during host cell traversal [11]. In all cases, parasites lacking individual PPLP proteins displayed a normal phenotype in asexual blood stages of the parasite lifecycle, indicating that members of this protein family are not involved in egress of blood stage merozoites.

Ookinetes are one of the two motile stages of the malaria parasite. The ookinete develops in the mosquito midgut from the round zygote over a period of ~ 15–18 h via an intermediate stage called the retort. After the establishment of apical polarity in the zygote [13], microtubules and the inner membrane complex (IMC) are gradually elongated to finally form the crescent shaped ookinete. The newly formed ookinete rapidly traverse the midgut epithelium. After reaching the basal side, the cell rounds up underneath the basal membrane and forms the oocyst, where protein synthesis and rounds of DNA replication leads to the formation of thousands of sporozoites after ~10 days. The sporozoites travel to the salivary gland, from where they are injected into a new host when the mosquito takes a new blood meal.

Recently, two studies were published which reported the phenotypic analysis of PPLP4 in *P*. *berghei* and *P*. *falciparum* [14,15]. In both cases, mutant parasites lacking the protein were unable to invade the midgut epithelium. Here we have created a null mutant of *P*. *berghei* PPLP4, and a detailed phenotypic analysis demonstrated the protein’s essential role in the ookinete for the traversal of the mosquito midgut.

## Materials and methods

### Ethics statement

All work was carried out in full conformity with Greek regulations consisting of the Presidential Decree (160/91) and law (2015/92) which implement the directive 86/609/EEC from the European Union and the European Convention for the protection of vertebrate animals used for experimental and other scientific purposes and the new legislation Presidential Decree 56/2013. The experiments were carried out in a certified animal facility license (EL91-BIOexp-02) and the protocol has been approved by the FORTH Committee for Evaluation of Animal Procedures (6740/ 8/10/2014) and by the Prefecture of Crete (license number # 27290, 15/12/2014).

### Parasite strains and parasitology methods

The *P*. *berghei* strains ANKA 2.34 was used in this study. The HPE line which does not form gametocytes has been described previously [16]. *P*. *berghei* parasites were maintained in *Theiler’s Original* mice. Infectivity of sporozoites was tested by feeding mosquitoes on C57/BL6 mice. Ookinetes were cultured in vitro as described [17]. Ookinete conversion rate (percentage of female gametes developing into ookinetes) was determined by labeling live cells from *in vitro* ookinete cultures with the monoclonal antibody 13.1 and anti-mouse Alexa-488 conjugated antibody. Ookinete motility assays were carried out as previously described [18]. Movies were analyzed with Fiji and the Manual Tracking plugin (http://pacific.mpi-cbg.de/wiki/index.php/Manual_Tracking). Mosquito infections were undertaken in *Anopheles gambiae* mosquitoes strain G3 and dissections for oocyst counts were done at 12 days post feeding. For injection of WT and *pplp4(-)*, *in vitro* cultured ookinetes (500 ookinetes in 69 nl) were injected in the thorax of CO_2_ anesthetized female mosquitoes using a Nanoject II hand held microinjector (Drummond). Salivary gland dissections took place on day 20 post injection. Salivary gland sporozoites were counted in the haemocytometer. To determine infectivity to mice, infected mosquitoes were allowed to feed on anaesthetized C57/BL6 mice at day 20 post injection. The infections were monitored by daily analysis of Giemsa-stained smears of tail blood.

### Generation of plasmid for gene disruption of the *P*. *berghei pplp4* locus, transfection and genotyping

The plasmid for the *pplp4* gene disruption by double cross over was constructed in the standard vector pL0001 (http://www.mr4.org). The left fragment encompassed bps 42 to 471 following the start codon, and the right fragment 1640 to 2025 bps of the *pplp4* ORF. The DNA fragments were amplified from *P*. *berghei* gDNA using the primers summarized in S1 Table. The two fragments were cloned into the KpnI and HindIII sites and the EcoRI and BamHI sites respectively. The plasmid was digested with KpnI and BamHI before transfection. After recombination by double crossover a 1211 bp fragment in the middle of the target gene was replaced by the TgDHFR/TS cassette (S3 Fig).

Parasites were transfected and cloned as described [19]. Correct integration was verified by PCR with the primers indicated (S1 Table).

### RT-PCR

Each sample of RNA from mixed blood stages was obtained from 100 μl of infected blood. Ookinete RNA was isolated from ookinete cultures equivalent to 100 μl infected blood. The oocyst samples were obtained by dissection of midguts from infected mosquitoes. Total RNA was isolated using the TRI reagent from Sigma according to the manufacturer’s instructions. The cells were pelleted at 500x*g*, and then resuspended in 500 ml of TRI; alternatively midguts were homogenized directly in the TRI reagent. cDNA was synthesized using the Thermoscript RT-PCR System Kit (Invitrogen) according to the manufacturers protocol. The PCR was performed using *Taq* polymerase (Promega). The primers Per4Frw1 and Per4Rev1 were used to amplify *pplp4* transcript (S1 Table) and primers specific for the ubiquitously expressed *gapdh* gene was used to verify the quality of the cDNA [20]. The primers for analysis of *pplp3* and *pplp5* derived transcripts are indicated in S1 Table.

For the normalization of cDNA between wt and pplp4(-) ookinetes, the cDNA was measured in Nanodrop and the samples were adjusted to equal concentration. Using the gapdh primers in a 25-cycle PCR program to avoid saturation, the quantity of cDNA that results in bands of equivalent density in an agarose gel was estimated.

### Generation of parasites expressing PPLP4::mCherry fusion

The plasmid used for the construct was pBAT-SIL6 [21]. A 2146 bp genomic fragment was produced by PCR using primers Per4_Fw_SacII and Per4_Rev_HpaI. The fragment was inserted into the plasmid after digestion with SacII and HpaI. Before transfection, the plasmid was opened at the unique restriction site NcoI to be inserted in the *pplp4* gene via a single crossover. Transfection was performed as described above, and genotyping with the primers 5FRper4, 3Rper4 and mcherryR confirmed the presence of the transgene, while the primer pair 5UTRFwper4/3UTRrevper4 was used to test for the WT genomic architecture.

### Antibodies

An antiserum recognizing PPLP4 was produced against the *P*. *falciparum* protein. To do this, a fragment encoding amino acid residues 150–254 was cloned into the vector pGEX6.1 and the protein expressed as a GST and 6xHis tagged version in *E*. *coli* SHuffle cells. Expressed protein was purified from inclusion bodies solubilized by sonication in 1.5% (w/v) sarkosyl. The second antiserum directed against PPLP4 has been described [15]. The 13.1 antibodies recognizing the Pbs21 ookinete surface protein and SOAP have been described [22,23]. The antibody against GAP50 was a kind gift from Dr Julian Rayner [24]. mCherry was detected with a rabbit polyclonal antibody from Abcam. Secondary antibodies were anti-mouse (Alexa-488, Alexa-555) and anti-rabbit (Alexa-488, Alexa-546) conjugated to Alexa Fluor (Invitrogen) or anti-rabbit conjugated to Cy-3 (Jackson Research).

### Immunofluorescence analysis (IFA)

Mature ookinetes from an O/N culture were purified using NH_4_Cl and then placed on poly-L-lysine-coated covers slips before fixation with 4% paraformaldehyde (PFA) in PBS for 15 min. The fixative was removed and the cells were permeabilized with 0.2% saponin in PBS for 3 min. Permeabilization with 0.5% Triton X-100 was also tested, but no labelling was detected after this treatment. In the case of mCherry antibody permeabilization with 0.5% saponin and 0.5% Triton gave satisfactory results. Primary antibodies were diluted in PBS with 5% normal goat serum. After incubation with secondary antibodies cells were mounted in Vectashield (Vector laboratories).

Midgut ookinetes were labeled according to [15]. Briefly, mosquito midguts were dissected 24 h after blood feeding, the blood bolus removed to PBS, and a sample spotted on glass slides and allowed to air dry followed by fixation with methanol for 10 min at -80°C. After blocking in the presence of 0.5% BSA, 0.01% saponin the sample was incubated with the antiserum and processed as above.

Samples were analyzed using a Zeiss LSM 510 confocal laser scanning microscope attached to a Zeiss Axioskop 2 plus microscope.

Alternatively, as indicated, a DeltaVision Elite deconvolution system (Applied Precision) was used. Z-stacks (0.15–0.2 μm steps) were deconvolved using the default settings in the SoftWoRx 5.0 acquisition software. 3D-Structured Illumination Microscopy (3D-SIM) was implemented on a DeltaVision OMX V4 Blaze™ (Applied Precision). Samples were excited using 488 and 568 lasers and imaged using 528/48 nm, 608/37 nm and with a 60x oil immersion lens (1.42 NA) as previously described [25]. Images were analyzed with ImageJ software (http://rsbweb.nih.gov/ij/).

### Electron microscopy analysis of *pplp4(-)* ookinetes

Ookinetes from an *in vitro* culture were purified using Nycodenz gradient centrifugation [17]. Nycodenz-enriched ookinetes were fixed in 2.5% glutaraldehyde, 2% paraformaldehyde, 2 mM CaCl2 in 0.1 M sodium cacodylate buffer, pH 7.4, overnight at 4°C, and processed according to Perry and Gilbert [26]. Parasites were washed in cacodylate buffer and post-fixed with 1% OsO4 in 0.1 M sodium cacodylate buffer for 1 hour at RT, treated with 1% tannic acid in 0.05 M cacodylate buffer for 30 min and rinsed in 1% sodium sulphate in 0.05 M cacodylate buffer for 10 min. Post-fixed specimens were washed, dehydrated through a graded series of ethanol solutions (30–100% ethanol) and embedded in Agar 100 (Agar Scientific Ltd, UK). Ultrathin sections, obtained by an UC6 ultramicrotome (Leica), were stained with uranyl acetate and Reynolds’ lead citrate and examined by a FEI/Philips EM 208S Transmission electron microscope equipped with acquisition system/Megaview SIS camera at 80kV.

### Midgut staining

At 24 h post-feeding, infected mosquitoes were dissected, the blood meal carefully removed and the midguts were fixed and permeabilized for 1 h with 4% PFA, 0.2% saponin in PBS. Subsequently midguts were washed in 0.2% saponin in PBS, blocked in 5% normal goat serum (Sigma-Aldrich) in PBS for 30 min. Ookinetes were labeled with the 13.1 antibody recognizing the surface protein Pbs21, mosquito cells were visualized with FITC conjugated phalloidin, and nuclei were stained with TO-PRO. Specimens were analyzed as described above.

### Western blot analysis

Blood from an infected mouse was diluted in ookinete culture medium and incubated at 19°C for 8 h. Half of the culture was then pelleted (retort sample) and red blood cells lysed with ammonium chloride. After another 16 h ookinetes were enriched in the same way. The two samples were processed similarly and in parallel. They were lysed by sonication in the presence of a cocktail of protease inhibitors (Sigma P8340) and 100 μΜ PMSF. The samples were then pelleted at 700xg for 10 min to remove unbroken cells. The samples were then centrifuged at 15 000xg for 10 min and the supernatant were processed for SDS-PAGE and analyzed by Western blot. The blots were probed with the monoclonal anti-mCherry antibody. The secondary anti-rabbit antibody was conjugated with horse radish peroxide. The signal was detected using the SuperSignal West Pico solution (Pierce Biotechnology).

## Results

### Perforin 4 is expressed in ookinetes

**Fig 1:**
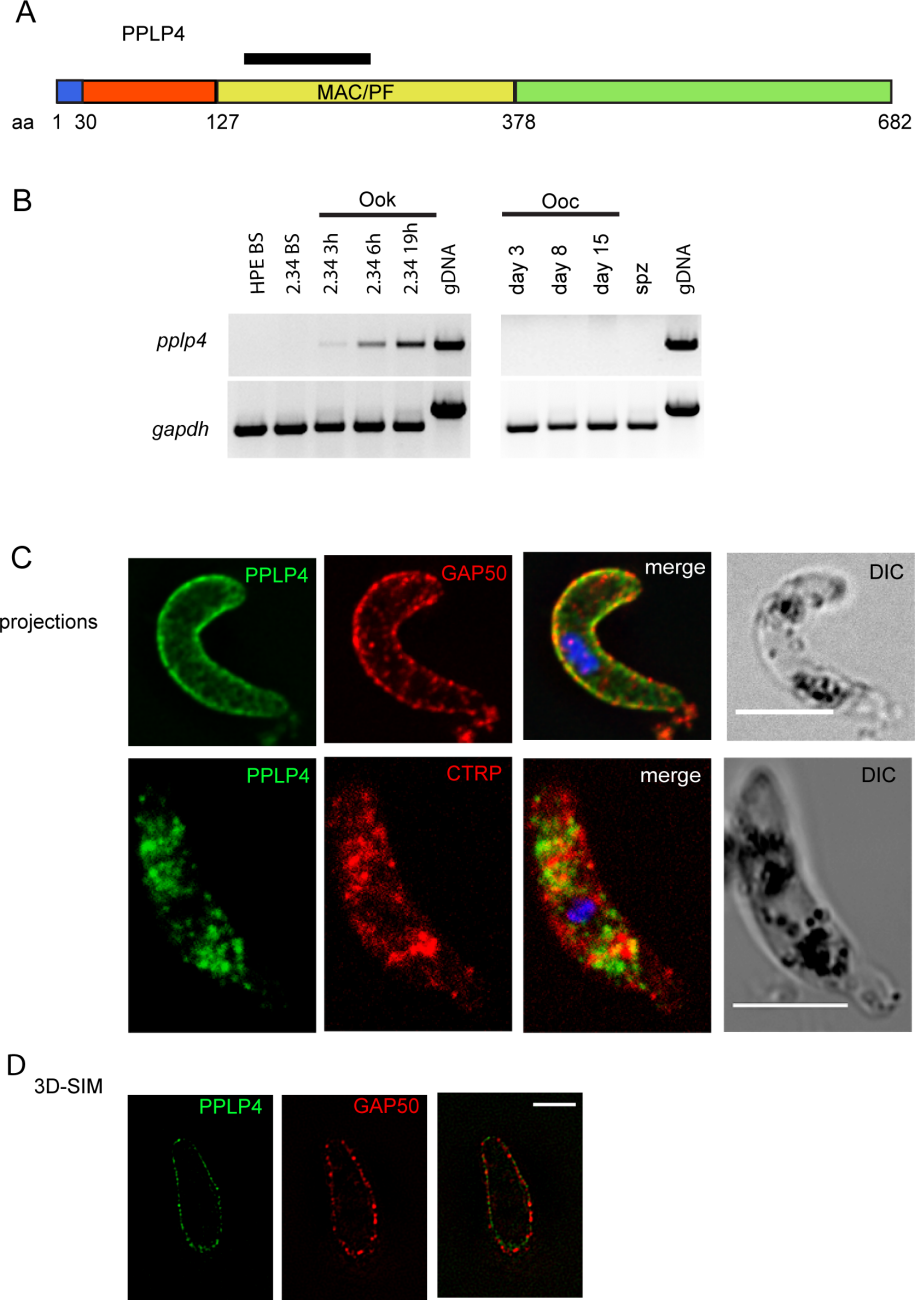
PPLP4 is expressed in ookinetes. A. Schematic representation of the PPLP4 protein. The different domains are indicated: signal peptide (blue), N-terminal domain (orange), MACPF domain (yellow), C-terminal domain (green). The number of amino acids of each domain is indicated below the figure. The fragment of the *P*. *falciparum* PPLP4 used to produce the antiserum is indicated with a black line above the protein. Numbers refer to the gene model of *P*. *berghei*. B. RT-PCR analysis of expression of the *P*. *berghei pplp4* gene. mRNA was derived from blood stages (BS) of HPE, a strain that does not form gametocytes, and ANKA 2.34, which forms normal gametocytes; Ook: samples obtained at 3, 6, and 19 h after seeding of an ookinete culture to follow expression of the mRNA during ookinete development; mRNA isolated from midguts containing oocysts (Ooc, at day 3, 8 and 15 after blood feeding) and salivary gland sporozoites (spz). Primers specific for the ubiquitously expressed *gapdh* gene were used to verify that all samples contained parasite cDNA in roughly equal amounts. C. Immunofluorescence analysis of *P*. *berghei* ookinete labeled with the antiserum against PPLP4 (green) and with a GAP50 antibody (red) (top row). DNA was labeled with DAPI (blue). The images are projections derived from a deconvolved stack. Bottom row shows a *P*. *berghei* ookinete expressing PPLP4 fused to mCherry, labelled with antibodies against mCherry (green) and the micronemal CTRP (red). DNA was labeled with DAPI (blue). The images are a projection of stacks obtained by confocal imaging. Scale bar 5 μm. D. Single section of an equatorial plane of an ookinete labeled with antibodies directed against PPLP4 (green) and GAP50 (red) imaged with 3D-Structured Illumination Microscopy (3D-SIM). Scale bar, 2 μm. The same ookinete is shown in S1 Movie.

In *P*. *berghei* PPLP4 is encoded by gene PBANKA_0711400 located on chromosome 7 in proximity to the gene encoding PPLP5, the two genes are separated only by the gene PBANKA_0711500 encoding a protein of unknown function. PPLP4 has so far been detected only in ookinetes [27]. The protein has an N-terminal putative signal peptide, suggesting that it is directed into the secretory system. The MACPF signature motif (Y/W)-X6-(F/Y)GTH(F/Y)-X6-GG is conserved, with one substitution (GTH(F/Y)) to (GTHV) that is found in all *Plasmodium* MACPF proteins (Fig 1A, S1 Fig). PPLP4 and PPLP5 both have short N-terminal domains preceding the model of Apicomplexan MACPFs (as defined in the model PTZ00482 at NCBI), while the other three *Plasmodium* perforin-like proteins have substantially longer distinct N-terminal domains. After removal of these divergent N-terminal sequences an alignment of all 5 PPLPs was obtained, showing that the five proteins are not very similar (S2 Fig). A block of 30 residues near the beginning of the aligned sequences is well conserved (green line), and as expected the domain containing the signature motif is also well conserved (blue line). Some of the amino acid residues that are conserved in the other four *Plasmodium* PPLPs (asterisks in S2 Fig) and suggested to be important for the structure [1] are not conserved in PPLP4. Notably there are six conserved Cys residues in the C-terminal domain of all the *Plasmodium* proteins, which are also found in *T*. *gondii* PLP1, and another five conserved in four of the five proteins.

We investigated expression of the *pplp4* gene by reverse transcription PCR (RT-PCR) analysis of mRNA derived from mixed blood stages of the ANKA 2.34 WT strain and the HPE line, which does not produce gametocytes. We also tested mRNA originating from an ookinete culture at 3, 6, and 9 h post seeding, representing zygotes and retort stages, and from mature ookinetes as well as oocysts and sporozoites. The results (Fig 1B) show that the *pplp4* gene is not transcribed in blood stages, with the transcript first detected at 3 h after the seeding of an ookinete culture, a time corresponding to zygote formation, and being continuously present until the mature ookinete stage. No expression was detected in oocysts or sporozoites.

To investigate expression of PPLP4 protein, we generated an antiserum recognizing a fragment of the *P*. *falciparum* PPLP4 (PF3D7_0819400), corresponding to amino acids 150–254 (marked in Fig 1A, black). The antiserum specificity was verified by labeling parasites lacking PPLP4 (S3A Fig). Immunolabelling of retorts, an intermediate form of the developing ookinete, derived from an ookinete culture 8 h after seeding, revealed the presence of the protein in a dotted pattern in the cytoplasm (S4A Fig), confirming the RT-PCR data. We used the same antiserum to examine mature ookinetes (Fig 1C top row) which revealed that the protein is localized close to the pellicle. Sections through the equatorial plane confirmed that PPLP4 is present in a punctate pattern close to the parasite periphery there was no signal in the cytoplasm of the cell (S4B Fig, f-j). In an effort to determine the nature of this compartment we dual labelled the samples with an antibody directed against the GAP50 protein which resides in the inner membrane complex [24]. The GAP50 signal also exhibited a punctate peripheral pattern (Fig 1C) indicating that the two proteins are located close to each other.

Having established that the proteins are in proximity we determined their spatial relation using 3D-Structured Illumination Microscopy (3D-SIM) to visualize PPLP4. This revealed that the punctate structures are smaller in size than is evident using conventional microscopy and also that GAP50 is located in very small punctate structures (Fig 1D, S1 Movie). The two proteins both localized to the periphery of the ookinete in non-overlapping compartments. These data indicate that PPLP4 is present in vesicular compartments that are delivered to the periphery.

Using a different antibody developed against *Pf*PPLP4 [15] revealed that the labelling pattern in *P*. *berghei in vitro* cultured ookinetes was consistent with our previous finding, showing a dotted peripheral pattern (S5A Fig). Similar results were obtained when we labeled midgut ookinetes using the protocol described [15] (S5B Fig).

To further investigate the trafficking and cellular localization of PPLP4, we generated a parasite mutant expressing PPLP4 fused C-terminally to mCherry. Surprisingly, in live ookinetes the chimeric protein was detected in the punctate structures in the parasite cytoplasm both in dots and diffusely towards the apical end (S4D Fig). The *pplp4*::*mCherry* mutant formed ookinetes with similar conversion rate to WT; oocysts were also formed and sporozoites transmitted to a naïve mouse in two subsequent experiments (S6 Fig).

Dual immunolabeling with antibodies directed against mcherry and the micronemal protein CTRP [28] showed that the punctate structure are distinct from the micronemes. (Fig 1C bottom row and S4C Fig).

In order to explain the absence of signal in the *pplp4*::*mCherry* ookinete periphery, we hypothesized that during protein export to the ookinete pellicle the mCherry tag at the C-terminal of PPLP4 may be processed leading to cleavage of the tag. To test this hypothesis we performed a Western blot analysis of *pplp4*::*mCherry* retorts and ookinetes using the anti-mCherry antibody. The results showed that two bands were recognized of molecular weight ~110 kDA (doublet) and 30 kDA that corresponds to the size of the chimeric protein and mCherry respectively. This supports the hypothesis that the C-terminus tag is cleaved during trafficking of PPLP4 to the periphery of ookinetes. Furthermore we measured by densitometry the ratio of the intensity of the two bands of each sample (intensity of 110 kDA /intensity of 30kDA) between retorts and ookinetes and a small increase of 30% was observed in the retorts (S3E Fig).

Taken together, these data indicate that PPLP4 is packaged into vesicular structures that are trafficked to the parasite periphery.

### Knockout mutants lacking PPLP4 form ookinetes but do not form oocysts

**Fig 2:**
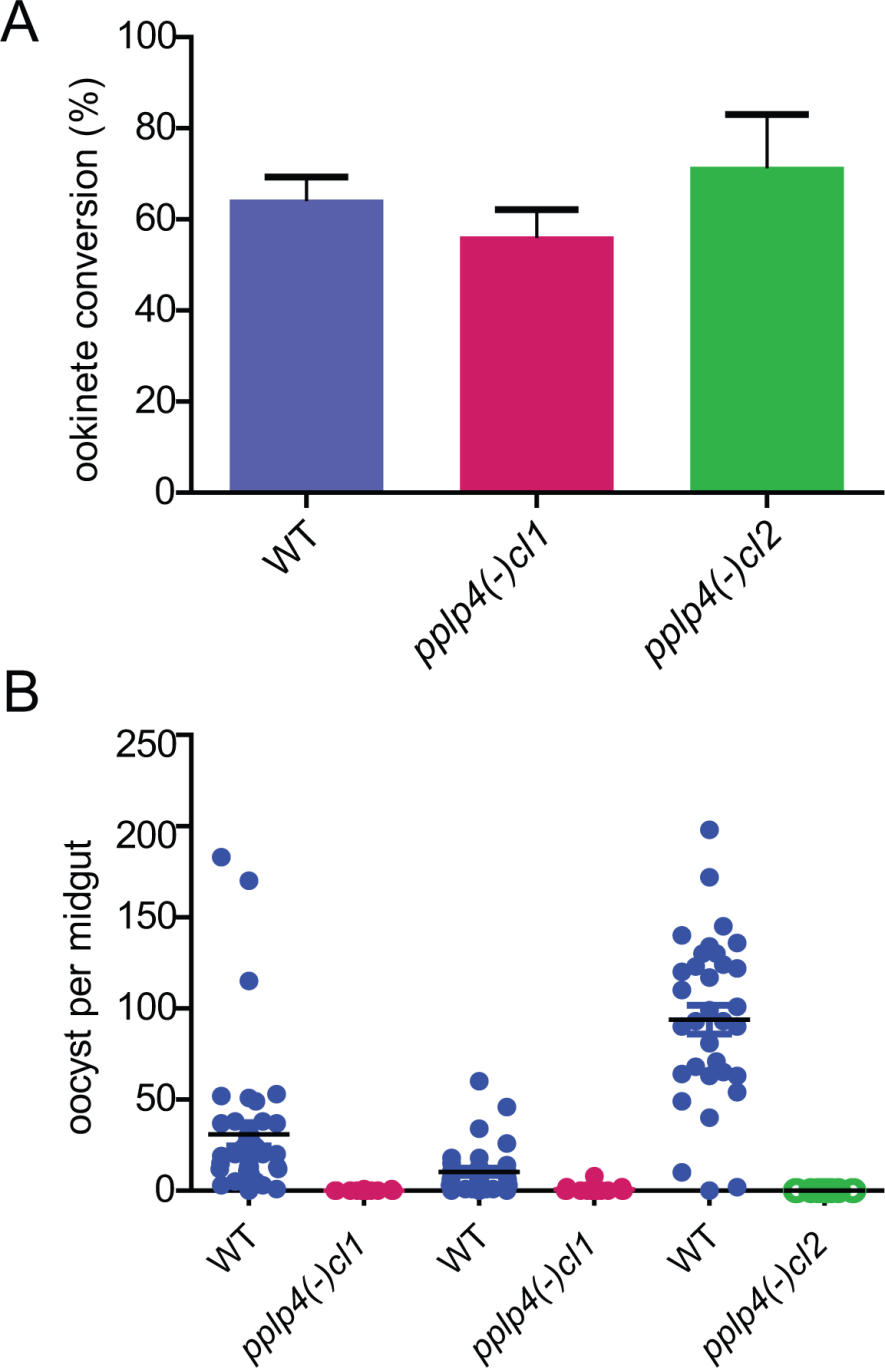
Parasites lacking PPLP4 are blocked in mosquito transmission. A. Ookinete conversion of *P*. *berghei* WT and *pplp4(-)* mutant clones 1 and 2. The graph shows data from 6 experiments of WT (blue) and *pplp4(-) cl1* (red) and four of *pplp4(-) cl2* (green). Error bars, S.E.M.. B. Oocyst counts from mosquitoes dissected 12 days after feeding of gametocytes from WT (blue) and the two *P*. *berghei* mutant clones 1 (red, 2 experiments) and 2 (green, 1 experiment). Three independent experiments are shown. Differences between WT and the mutants are significant. *** P<0.0001, Kruskal-Wallis non-parametric test.

The *P*. *berghei pplp4* gene was disrupted by introducing the antifolate resistance cassette encoding *T*. *gondii* DHFR/TS via double cross over homologous integration into the middle of the gene (S3B Fig). Clones were obtained by limiting dilution and verified by PCR and Southern blotting of genomic DNA (S3C and S3D Fig). Two clones (clones 1 and 2) were analyzed in parallel. To verify the loss of the protein, ookinetes were probed with the antiserum, and, as expected, no signal was detected (S3A and S5 Figs). The mutation had no detectable effect on asexual proliferation and the numbers of gametocytes formed were roughly equal to the WT and showed a similar ratio of males to females (WT males 3.1±0.1%, females 3.2±0.6%; *pplp4(-)* males 2.2±0.5%, females 2.2±0.2%; average of three experiments, values ± S.E.M), in concordance with the lack of protein expression in these stages. *In vitro* ookinete conversion was similar to WT (Fig 2A).

Next, we determined the ability of the mutant parasites to colonize mosquitoes. After feeding the *P*. *berghei pplp4(-)* mutant to *A*. *gambiae* mosquitoes very few oocysts were detected in three independent experiments (two for *pplp4(-) cl1* and one experiment using *pplp4(-)cl2*) (Fig 2B) and the mutant parasites were unable to transmit to naïve mice; this experiment was also repeated three times (data not shown). These results strongly suggested a defect in ookinete to oocyst conversion.

### *pplp4(-)* ookinetes display no gross abnormalities in morphology

**Fig 3:**
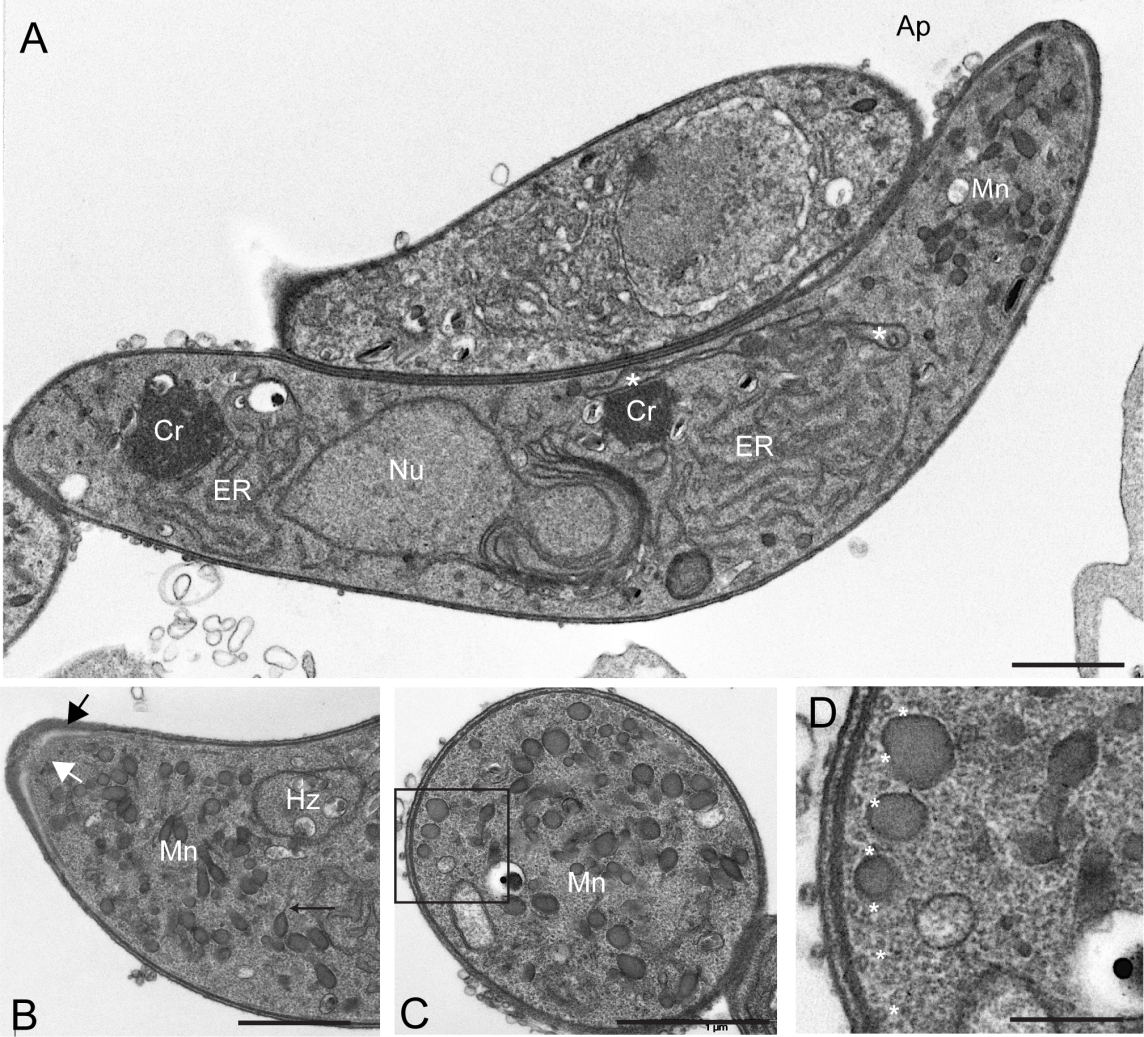
Transmission EM analysis of *pplp4(-)* ookinetes does not reveal major morphological abnormalities. A. Section through the middle of an ookinete. Nucleus (Nu), endoplasmic reticulum (ER), and crystals (Cr) are clearly visible, as well as micronemes (Mn) in the apical end (Ap) of the cell. The elongated mitochondrion is indicated with white asterisks. Another ookinete sectioned at an oblique angle is also visible. Scale bar, 2 μm. B. Higher magnification of the apical part of another ookinete. Pear shaped micronemes, some with a stalk (thin black arrow) as well as the electron dense collar with the underlying ring (black arrow) and the aperture (white arrow) are seen. Hz, hemozoin. Scale bar, 1 μm. C. Cross section through an ookinete revealing micronemes (Mn) and the pellicle. Scale bar, 1 μm. D. Magnification of the boxed part in C. The three layered pellicle with regularly spaced microtubules (black asterisks) and micronemes is visible. Scale bar, 0.25 μm.

To determine whether the *pplp4(-)* ookinetes displayed any gross morphological defects we examined them by transmission EM analysis of ultrathin sections (Fig 3). No major abnormalities were detected (Fig 3A). Many micronemes were present in the area between the nucleus and the apical end of the parasite, and some displayed the typical pear-shape and stalk (Fig 3A and 3B). The ultrastructure of the apical prominence was clearly visible, characterized by the typical conical structure with an electron dense collar, in close contact with the inner membrane, and a central aperture limited by the plasma membrane (Fig 3B). Beneath the collar there was the apical ring, a less electron dense layer, subtended by the subpellicular microtubules. These were regularly spaced as shown by cross sectional imaging (Fig 3C and 3D) and the inner membrane complex was clearly outlined (Fig 3C and 3D). We also used the validated antibody [22] against the micronemal SOAP protein to determine if this protein was present in the mutant and with a similar localization as in the WT. These experiments did not reveal any major differences in localization or signal intensity of the SOAP protein (S7 Fig). We conclude that the loss of PPLP4 does not result in gross abnormalities in the ookinetes that could explain the failure of the mutant to form oocysts.

### *pplp4(-)* ookinetes are motile but do not invade the epithelium

**Fig 4:**
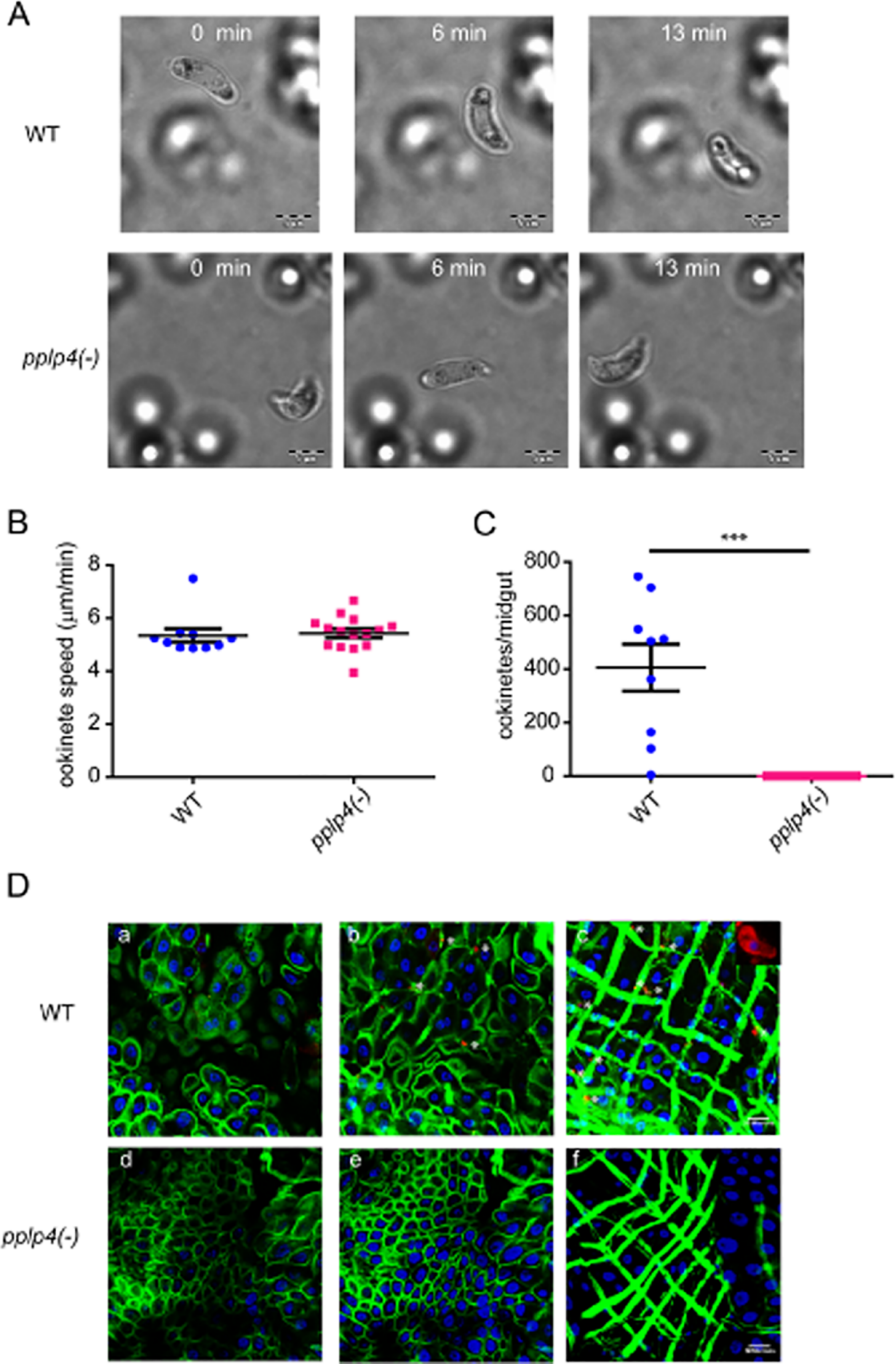
*P*. *berghei pplp4(-)* ookinetes are normally motile but do not invade the midgut epithelium. A. Representative frames of time lapse videos, at 0, 6 and 13 min after start of capture (S2, S3 movies). Top, WT, Bottom, *pplp4(-)*. Scale bars, 5 μm. B. The speed of WT (n = 10) and *pplp4(-)* (n = 14) ookinetes was measured from time lapse movies. The time lapse videos were captured at 1 frame/5 sec, and the total length of the movies used for analysis was 20 minutes. There was no significant difference comparing the two strains (Student’s t-test). C. Ookinetes counted in midguts dissected from mosquitoes 24 h after an infected blood meal. WT, n = 9, *pplp4(-)*, n = 20. Not a single ookinete was detected in the midguts fed with the *pplp4(-)* mutant. ***, P<0.0001, Student’s t-test. D. Selected sections of confocal stacks of mosquito midguts infected with WT (a-c) and *pplp4(-)* (d-f) parasites. a and d, sections from the midgut close to the lumen, b and e, from the middle of the epithelial cells and c and f from the basal side. Inset in panel c shows a magnification of a WT ookinete in the midgut. Ookinetes were labeled with the 13.1 antibody recognizing the Pbs21 surface antigen (red) and are indicated by asterisks. The midguts were visualized using FITC conjugated phalloidin, labeling mosquito actin (green) and TO-PRO staining nuclei (blue). Scale bar, 50 μm.

One possible explanation for the block in ookinete to oocyst development is that ookinete motility was affected in the mutant. To compare motility of *in vitro* cultured WT and *pplp4(-)* ookinetes, they were mixed with Matrigel and time lapse movies imaged (Fig 4A and S2 and S3 Movies). The speed of ookinete migration was found to be similar for both WT and mutant ookinetes with mean averages of 5.3 μm/min and 5.4 μm/min respectively (Fig 4B). These values are very close to the 6 μm/min value previously reported for WT parasites [18,29,30].

We next performed experiments to determine whether the *P*. *berghei pplp4(-)* ookinetes were associated with the midgut epithelium in infected mosquitoes. Mosquitoes were allowed to feed on mice infected with WT and mutant parasites and the midguts were dissected 24 h post feeding. Epithelium-associated ookinetes were labeled with an antibody recognizing the surface protein Pbs21. The results revealed that in the WT-infected midguts hundreds of ookinetes could be detected in each midgut, as expected. However, no *pplp4(-)* parasites were detected associated with the epithelium (Fig 4C and 4D).

### Mutant ookinetes develop in the midgut but remain in the food bolus

**Fig 5:**
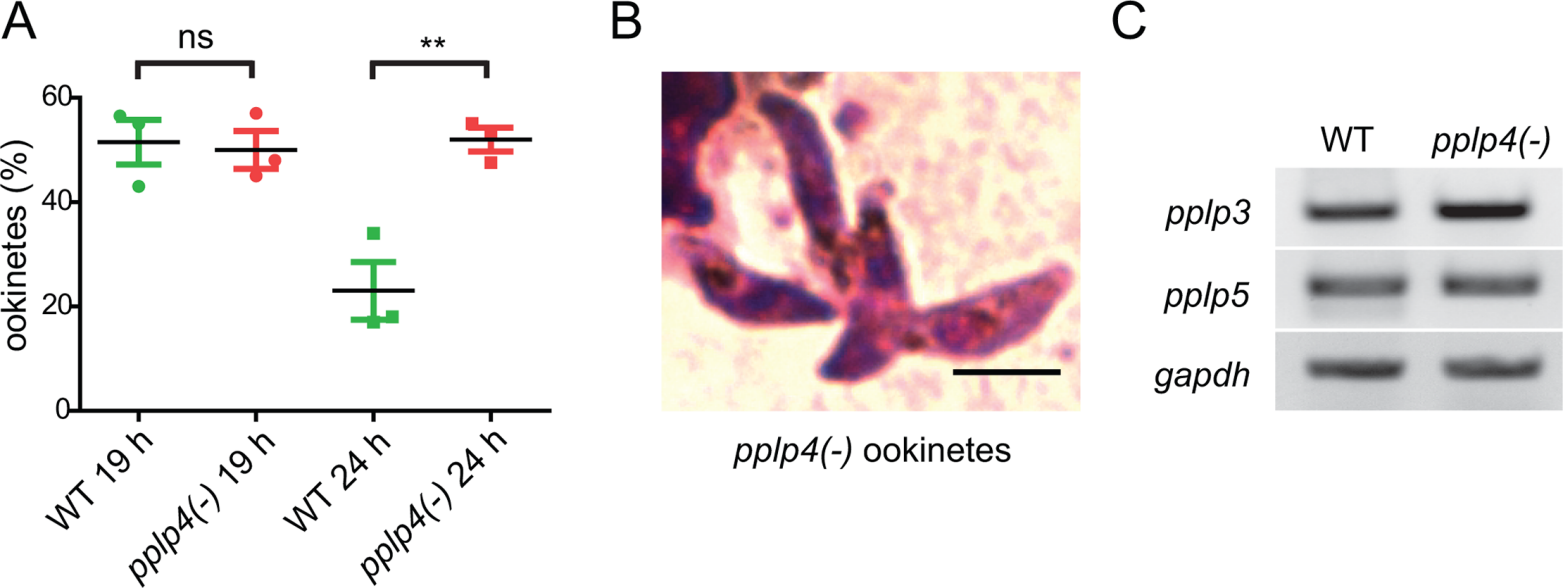
*pplp4(-)* ookinetes are formed in the mosquito midgut but remain in the food bolus. A. Percentage ookinetes detected in the midguts 19 h (circles) and 24 h (squares) after feeding mosquitoes on mice infected with WT (green) and mutant parasites (red). The number of ookinetes was normalized against gametocytes, gametes, zygotes and ookinetes in the same sample. The cells were counted on Giemsa stained smears of the blood bolus. In each experiment six midguts were scored, and the experiments were repeated three times. ns, non-significant; ** P<0.01 Student’s t-test. B. Representative *pplp4(-)* ookinetes from the Giemsa stained smears of the blood bolus from an infected mosquito 19 h after the blood meal. Scale bar, 5 μm. C. RT-PCR analysis of cDNA derived from WT and *pplp4(-)* mutant using primers specific for *pplp3* and *pplp5* (30 cycles in each case). The cDNA was normalized using primers specific for the ubiquitously expressed *gapdh* at 25 cycles to avoid saturation.

To verify that the failure to detect ookinetes in the midgut epithelium was due to a block in invasion and not a result of blocked formation of ookinetes we wished to determine whether ookinetes were indeed formed in the mosquito midgut. Mosquitoes were allowed to take a blood meal on WT and *pplp4(-)* infected mice. 19 h and 24 h post feeding, midguts were dissected, the blood bolus was removed and smeared on slides, which were then stained with Giemsa. Gametocytes, gametes, zygotes and ookinetes were counted and the number of ookinetes was normalized to the total number of these stages. At 19 h in both the WT and *pplp4(-)* infected midguts, ookinetes constituted roughly 50% of parasite cells (Fig 5A). Importantly, the mutant ookinetes had a normal appearance as determined from the Giemsa stained smears (Fig 5B). 5 h later, when the WT ookinetes normally invade the midgut epithelium the percentage of WT ookinetes had, as expected, decreased significantly. The mutant ookinetes however, remained in the blood bolus in similar numbers to the sample at 19 h. We conclude that the mutant parasites develop into morphologically normal and motile ookinetes but these are unable to escape from the blood bolus.

Both PPLP3 and PPLP5 are also important for ookinete invasion of the midgut epithelium. To exclude the possibility that the phenotype of the *pplp4(-)* parasites may be due to a down-regulation of expression of the genes encoding these other two perforins we performed RT-PCR on samples derived from WT and mutant ookinetes using primers specific for the two genes (Fig 5C). The results did reveal no major change in transcription of either gene in the *pplp4(-)* mutant. Antibodies directed against PPLP3 and PPLP5 were not available to test whether there were any differences in the amount of the proteins or in their localization in the cell.

### *pplp4(-)* sporozoites are infectious to naïve mice

In view of the apparent block in traversal of the midgut epithelium in the *pplp4(-)* mutant, further experiments were performed to determine whether *pplp4(-)* ookinetes were able to develop into sporozoites when injected directly into the mosquito thorax, thus by-passing the midgut epithelium. Salivary glands were dissected 20 days after injection of *in vitro* cultured WT and *pplp4(-)* ookinetes. Roughly equal numbers of sporozoites were recovered from mosquitoes injected with the two strains (Experiment 1: WT 2.2x10^3^ sporozoites/mosquito (n = 9); *pplp4(-)* 1.2x10^3^ sporozoites/mosquito (n = 15). Experiment 2: WT 1.6x10^3^ sporozoites/mosquito (n = 21), *pplp4(-)* 1.9x10^3^ sporozoites/mosquito (n = 30)). We next tested whether the mutant sporozoites were infective to a mouse by bite. In experiments testing the two *pplp4(-)* clones 17 and 11, the mouse became positive for parasites on day 5. Genotyping of the parasites in the mouse infected with the *pplp4(-)* mutant sporozoites confirmed that the mutant had been transmitted and there was no contamination with WT parasites (S8 Fig). Taken together these results show that PPLP4 is required for midgut invasion, but parasites lacking the protein are able to complete mosquito development and infect mice if the midgut barrier is by-passed.

## Discussion

We determined the expression of the perforin-like protein 4 in the rodent model parasite *P*. *berghei*. *Pplp4* transcript was only detected in developing and mature ookinetes. It was first detected 3 h after seeding of an ookinete culture and the protein was detected in retorts 8 h after seeding. We used two different antisera raised against the *P*. *falciparum* PPLP4 to detect the protein, neither of which recognized the protein in the *pplp4(-)* mutant.

PPLP4 was detected as punctuate structures in the parasitic cytoplasm of retorts and subsequently delivered close to the parasite periphery in the mature ookinete, as indicated by the close association (but not overlap) in the dual labeling experiments using the antiserum against *Pf*PPLP4 and the well-characterized GAP50 antibody in 3D SIM super resolution microscopy.

We created a mutant expressing a chimeric PPLP4 fused to mCherry. We confirmed that this mutant was not impaired in any of the stages of the mosquito and also transmitted to naïve mice. In this mutant punctate labeling was observed both in the cytoplasm and the periphery of the ookinetes. The extensive accumulation of the vesicles in the cytoplasm may indicate that the mCherry fusion decreases the efficiency of trafficking of PPLP4 to the region of the periphery.

Similar differences in cell localization of a perforin protein were observed in *P*. *falciparum* sporozoites [12]. In some sporozoites PPLP1 was detected in the cytoplasm, co-localized with the micronemal protein PfAMA1, while in others the protein was found at the periphery suggesting that PPLP1 is a micronemal secreted protein that is trafficked to the parasite surface in order to interact with the target membrane.

In our experiments the lack of co-localisation of PPLP4 with CTRP did not allow us to draw any safe conclusions regarding the micronemal localization of PPLP4, although it is possible that the two proteins reside in distinct micronemal subsets as has been described in *T*. *gondii* [31]. Taken together we suggest that PPLP4 is packaged to vesicles in the cytoplasm before being trafficked to the pellicle.

The absence of signal in the cytoplasm when using the PPLP4 antiserum may be explained if it cannot recognize the protein before it has been trafficked to the pellicle, for example if cytoplasmic PPLP4 is in a complex with other proteins or its conformation in this compartment is different from when it is present on the surface. On the other hand the absence of fluorescent signal from the periphery of the *pplp4*::*mcherry* mutant can be explained by the cleavage of the mCherry tag during PPLP4 export. We hypothesized that the mCherry in the C-terminal of PPLP4 is processed during trafficking of the protein to the ookinete pellicle. To investigate this a Western blot experiment was performed which revealed 30 kDa mCherry peptide in cell extracts of retorts and ookinetes and thus supporting this notion. Interestingly, cleavage of 12–20 amino acids at the C-terminus is necessary for activation human and mouse perforin [32].

Our results are different from those obtained in a study of the PPLP4 homolog in *P*. *falciparum* [15]. In that study PPLP4 was detected in a single compartment in the *P*. *falciparum* ookinete. We used the same antiserum and the same labelling protocol to probe *P*. *berghei* ookinetes resulting in a dispersed punctate peripheral location observed for both midgut and *in vitro* cultured ookinetes. We suggest that the PPLP4 containing vesicles may coalesce into a larger cisternal compartment in *P*. *falciparum*, while the vesicles remain unfused in *P*. *berghei*. However we cannot exclude that this may be due to variations in protein localizations among different ookinetes something which we have observed for micronemal proteins, for example when comparing different cells labelled for SOAP or CTRP or it reflects distinct localization of the protein in the two species.

A detailed phenotypic analysis of two independent knock-out mutant clones revealed that ookinetes were formed, but these were unable to develop into oocysts. These results are in complete consistence with those reported very recently in independent studies of *pplp4* in *P*. *berghei* and *P*. *falciparum* [14] [15]. We further showed that this was not due to abnormalities in the ookinete. We investigated cellular organization using transmission EM and tested motility *in vitro*, which revealed no differences to WT. Our combined data thus strongly suggest that the function of PPLP4 is related to the penetration of the epithelial cells of the midgut.

We also determined whether the protein had any function in oocyst development or sporozoite infectivity. We found that *in vitro* cultured mutant ookinetes formed sporozoites when the midgut epithelium was by-passed by injecting ookinetes directly into the mosquito thorax, and the resulting sporozoites were readily transmitted to mice by an infectious bite. Taken together, our results suggest that the function of PPLP4 is restricted to midgut invasion, although the possibility that PPLP4 could have a non-essential role in mosquito-mouse transmission cannot be ruled out.

Previous studies have revealed that two perforins have roles in the ookinete invasion of the midgut epithelium, namely PPLP3 or MAOP [10] and PPLP5 [8]. PPLP3 was shown to localize to the micronemes, while the localization of PPLP5 is unknown. Both function in destabilizing the host cell during entry, as mutants lacking either of these proteins are unable to invade the epithelial cells of the midgut and are trapped on the luminal side of the epithelium. Consequently, an almost complete block of oocyst development was observed in both these knockouts. One possible explanation for the result of the phenotype of the *pplp4(-)* mutant could be that the loss of PPLP4 leads to down-regulation of the other two perforins. RT-PCR analysis of the mutant with primers specific for PPLP3 and PPLP5 did not reveal gross differences in transcript abundance and thus this possibility is less likely.

In conclusion, the parasite uses three different MACPF containing proteins to invade the midgut epithelial cell, raising the possibility that the three proteins have different but related functions. The three perforin-like proteins could act in a complex, similar to what is found in mammalian complement [3], a notion that was also put forward in the aforementioned two studies [14] [15]. Prominent and well-studied MACPF proteins in mammals are components of the complement cascade. The proteins of the complement C5, C6, C7, C8α, C8β and C9 proteins all contain MAC domains, and interact to form a complex through ordered interactions. C5, C6 and C7 bind sequentially to the inner leaflet of the target membrane and function as a receptor for C8, which inserts into the membrane. The transmembrane pore is formed by oligomerization of C9 recruited by interaction with the C5-C8 complex. The diameter of the pore is roughly 10 nm, depending upon the number of C9 subunits forming the pore [3]. One could thus envisage that PPLP3, PPLP4 and PPLP5 may form a similar complex, and if so loss of one protein may affect the stability of other components of the complex. Due to a lack of antibody reagents we were unable to address that question in this study, but future experiments will address this question biochemically, similar to the published study of *T*. *gondii* [5].

What could be the function of pore formation in the mosquito midgut epithelial cells? MACPF-containing proteins mediate diverse functions, and in the case of complement the outcome is dependent on the number of pores in the target cell membrane and the type of cell attacked [3]. However, one possible outcome of pore formed by complement C5-C9 is the entry of Ca^2+^ leading to apoptosis. In the case of perforin, it has been suggested that granzyme passes through the pore resulting in apoptosis due to protease activity [2]. It is well known that after ookinete invasion the midgut epithelial cells undergo cell death [33,34], with features of apoptosis. It is tempting to speculate that the *Plasmodium* perforin-like proteins have a role in promoting apoptosis-like cell death, thus facilitating ookinete passage through the epithelium.

Another explanation for the presence of the three perforins in midgut invasion is that they have different roles. Our experiments showed that the *pplp4(-)* ookinetes remained in the blood bolus and it is thus possible that PPLP4 functions in the passage through the peritrophic membrane (PM), a structure consisting of chitin, proteins and proteoglycans which surrounds the blood bolus [35] and which has been shown to act as a partial barrier to ookinete invasion [36]. The *maop* mutant ookinetes were found to adhere to the apical membrane of the epithelial cells suggesting that they successfully crossed the PM. The mutant lacking PPLP5 was shown to have crossed the PM [8]. Here, we did not directly address the question whether the *pplp4(-)* parasites could pass through the PM and therefore this aspect remains to be investigated.

Given that the three PPLPs involved in ookinete invasion are predicted to form similar structures, they may present a combined target for transmission blocking strategies, including a vaccine, as the proteins are predicted to be secreted. The fact that each of the three proteins is essential would minimize the risk of selection of mutations that would result in parasites able to overcome the block in transmission.

## Supporting information

S1 Fig **Alignment of the predicted full-length *P*. *berghei* (top) and *P*. *falciparum* (PF3D7_0819400) PPLP4 proteins.** The two proteins are 52% identical, and 69% similar. Black shading denotes identity, while gray shading indicates conservative substitutions. The red bar denotes the secretory signal sequence (aa 1–30 in *P*. *berghei*, aa 1–28 in *P*. *falciparum*). The green bar indicates the MAC/PF domain (PFAM IPR020864), aa 127–386 in *P*. *berghei*, aa 120–370 in *P*. *falciparum*. The signature motif (Y/W)-X6-(F/Y)GTH(F/Y)-X6-GG is indicated with a blue bar below the sequence. One substitution GTH(F/Y) to GTHV is the only discrepancy with the signature motif, but all five *Plasmodium* perforins have a substitution to I, L or V at this position. An NCBI CD search retrieved no conserved domain or architecture when searched with the C-terminal domain aa 387–682. The alignment was performed using ClustalW (http://www.ebi.ac.uk/Tools/msa/clustalw2/) and visualization using Boxshade (http://www.ch.embnet.org/software/BOX_form.html).
(TIF)

S2 Fig Multiple sequence alignment of the five PPLPs of *P*. *berghei*. The N-termini were not included as indicated. PlasmoDB accession numbers and included aa region: PPLP1, PBANKA_1006300, aa 195–850; PPLP2, PBANKA_1432400 aa 349–999; PPLP3, PBANKA_0824200, aa 209 to 815; PPLP4, PBANKA_0711400, aa 33–682; PPLP5, PBANKA_0711600, aa 38 to 687. Green line, block of highly conserved region of unknown function; blue line, MACPF signature; orange arrowheads delineates the MACPF domain (Pfam01823); asterisks, conserved aa which have been recognized to be important for MACPF structure [1]; green arrowhead, conserved Cys residues, yellow arrowheads Cys residues conserved in four of the aligned sequences. Lines and asterisks are positioned below the alignment, arrowheads above. Alignment was performed as in S1 Fig.
(TIF)

S3 Fig Targeted disruption of the *pplp4* gene in *P*. *berghei*. A. Immunolabeling of WT (top) and *pplp4(-)* (bottom) ookinetes using the PPLP4 antiserum. The WT displays the dotted label typical of PPLP4 while no signal was detected in the mutant. The two pictures were obtained from experiments conducted in parallel and pictures were taken with the same settings. Inset: the picture of *pplp4(-)* enhanced to show the ookinete. Scale bar, 5 μm. B. Schematic representation of the WT genomic locus (WT), the fragment introduced to target the gene (pPPLP4) and the locus after integration resulting in the disruption of the ORF (*pplp4(-)*). Primers and the sizes of fragments obtained are indicated in red, EcoRI sites as green arrowheads and restriction length fragments in green font. C. Genotyping using the primers depicted in A for PCR of gDNA from WT and *pplp4(-)* The sequence of the primers is available in S1 Table. D. Southern blot of genomic DNA of *pplp4(-)* and WT gDNA digested with EcoRI. The probe corresponds to 461 bp of the 5’-FR and the complete ORF. Asterisks indicate positive bands. E. Western blot analysis of *P*. *berghei pplp4*::*mCherry* 8h retorts and ookinetes extracts. The two samples were derived from the same culture and they were processed in parallel (see Materials and Methods). The blot was probed with an antibody recognizing mCherry. A processed form of the chimeric protein is also detected in both samples (arrow). Molecular weights are indicated on the left.
(TIF)

S4 Fig Comparison of PPLP4 localization in WT and *pplp4*::*mCherry* parasites. A Immunofluorescence analysis of *P*. *berghei* retorts labeled with the antiserum against PPLP4 (green) (top row). Bottom row shows a *P*. *berghei pplp4*::*mCherry* retort labelled with mCherry antibody (green). DNA was labeled with DAPI (blue). Scale bar 5 μm. Images show detection of PPLP4 as early as 8h retort stage. B. Montage of single sections of the same WT ookinete shown in Fig 1C, top row, labeled with antibodies against PPLP4 and GAP50. C. Montage of single sections of the same *pplp4*::*mCherry* ookinete shown in Fig 1C, bottom row, labeled with mCherry and CTRP antibodies. Nuclei stained with DAPI (blue). Scale bar 5 μm. D. Live images of *pplp4*::*mCherry* ookinetes. Variations in protein localization between different cells were observed. In some ookinetes, PPLP4::mCherry is observed as dispersed punctuate structures in the cytoplasm while in others it is also observed in the apical end (arrow).
(TIF)

S5 Fig Immunolabeling of WT and *pplp4(-)* ookinetes with the antiserum against *Pf*PPLP4 described in Wirth *et al*. A. *In vitro* cultured ookinetes. Top row, WT; bottom row, *pplp4(-)*. B. Midgut ookinetes labeled using the same protocol as Wirth *et al*. Top row, WT; bottom row, *pplp4(-)*. The nucleus is highlighted in blue. Scale bars, 5 μm.
(TIF)

S6 Fig *pplp4*::*Cherry* phenotypic analysis. A. Genotyping with primers 5UTRFwper4/mcherryrev confirmed the presence of *pplp4*::*mCherry* parasites in oocysts (lane 1) and mutant sporozoites were able to infect a naïve mouse (lane 2). As controls genomic DNA from the transfectant *pplp4*::*mCherry* population and WT parasite (lanes 3 and 4 respectively) was used (top row). The quality of genomic DNA and the presence of WT parasites were tested using the primer pair 5UTRFwper4/3UTRrevper4 (bottom row). B. Ookinete conversion of *pplp4*::*mCherry* parasites similar to WT parasites. Values are average of three independent experiments. C. Oocyst formation of WT and *pplp4*::*mCherry* parasites was similar. Two independent experiments were carried out.
(TIF)

S7 Fig Micronemal SOAP is similarly localized in *pplp4(-)* mutant and WT ookinetes. Ookinetes were labeled with the antibody directed against SOAP. Images were obtained in an epifluorescence microscope without deconvolution. 6 *pplp4(-)* mutant (top row) and 7 WT (bottom row) ookinetes are shown to illustrate the individual differences in SOAP localization in ookinetes. Scale bar, 5 μm.
(TIF)

S8 Fig Genotyping of parasites transmitted by bite-back to naïve mice. The mosquitoes had been infected by injection of ookinetes into the mosquito thorax. Lanes 1, 3 and 5 genomic DNA from *pplp4(-)* cl 17 bite-back mouse. Lanes 2 and 4, gDNA from *pplp4(-)* cl 17 Lane 6. WT gDNA. Primers in Lane 1 and 2 were 5FRper4/ L695testing for left integration of the gene replacement, in Lane 3 and 4 DHFF/3Rper4 (right integration). WT contamination was tested using primers Per4fw1536/3Rper4.
(TIF)

S1 Movie 3D-SIM animation of ookinete labeled with antiserum directed against PPLP4 (green) and GAP50 (red). (AVI)

S2 Movie Motility of a WT ookinete. (AVI)

S3 Movie Motility of a *pplp4(-)* ookinete. (AVI)

S1 Table Primers used in the construction and genotyping of the *pplp4(-)* disruptionmutant and the *pplp4*::*mCherry* mutant as well as the RT-PCR detection of *pplp3* and *pplp5* transcripts. (DOCX)
