# Correction: Essential role of *Plasmodium* perforin-like protein 4 in ookinete midgut passage

**DOI:** 10.1371/journal.pone.0204083

**Published:** 2018-09-12

**Authors:** Elena Deligianni, Natalie C. Silmon de Monerri, Paul J. McMillan, Lucia Bertuccini, Fabiana Superti, Maria Manola, Lefteris Spanos, Christos Louis, Michael J. Blackman, Leann Tilley, Inga Siden-Kiamos

The following information is missing from the Funding section: This study was supported by the Francis Crick Institute, which receives its core funding from Cancer Research UK (FC001043), the UK Medical Research Council (FC001043), and the Wellcome Trust (FC001043).
