# Supplementary figures and images for: Essential role of Plasmodium perforin-like protein 4 in ookinete midgut passage
Source: PLoS One. 2018 Aug 13;13(8):e0201651. doi: 10.1371/journal.pone.0201651 (PMC6089593; doi:10.1371/journal.pone.0201651)

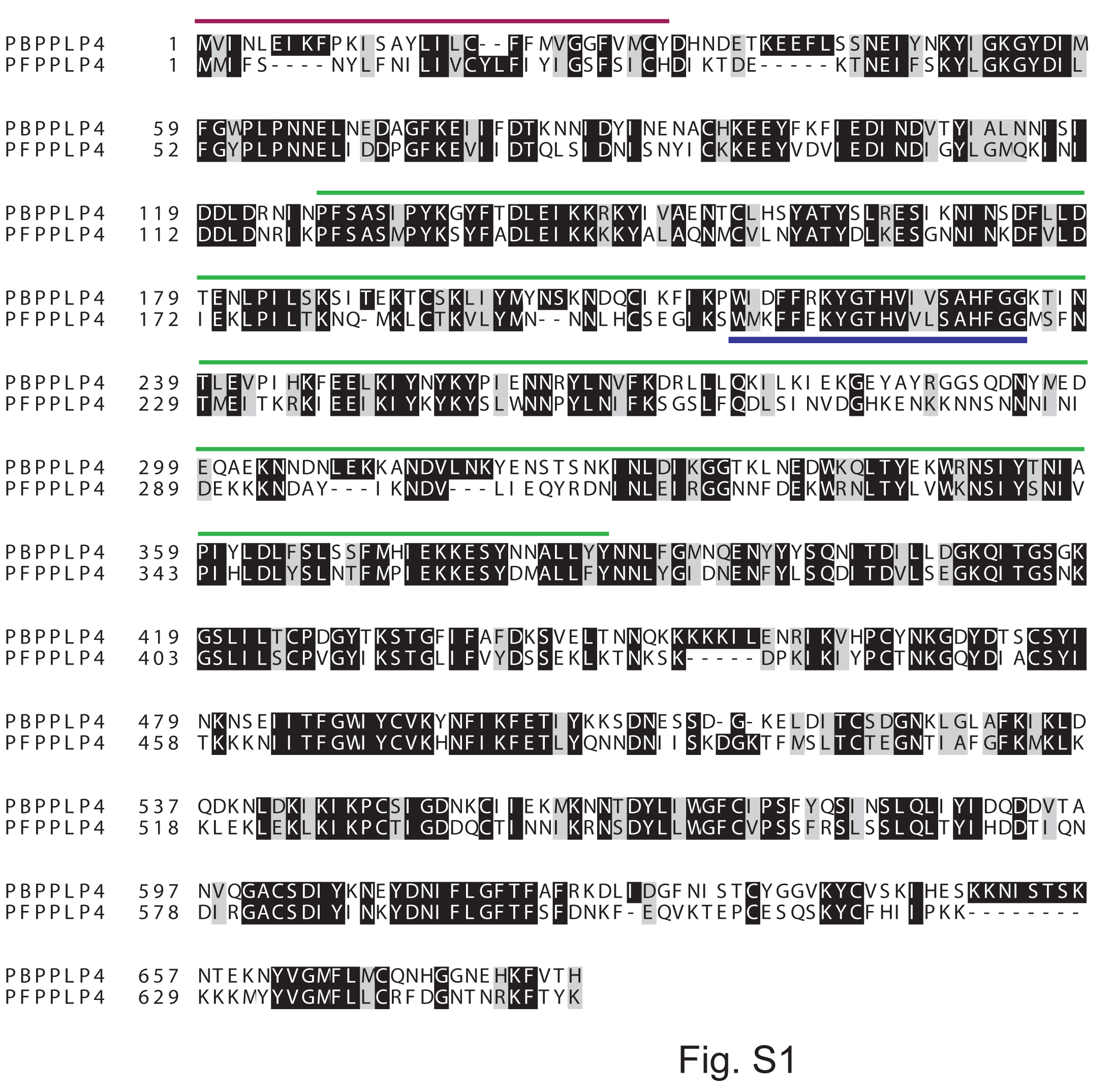

Supplement: S1 Fig — Alignment of the predicted full-length P. berghei (top) and P. falciparum (PF3D7_0819400) PPLP4 proteins. The two proteins are 52% identical, and 69% similar. Black shading denotes identity, while gray shading indicates conservative substitutions. The red bar denotes the secretory signal sequence (aa 1–30 in P. berghei, aa 1–28 in P. falciparum). The green bar indicates the MAC/PF domain (PFAM IPR020864), aa 127–386 in P. berghei, aa 120–370 in P. falciparum. The signature motif (Y/W)-X6-(F/Y)GTH(F/Y)-X6-GG is indicated with a blue bar below the sequence. One substitution GTH(F/Y) to GTHV is the only discrepancy with the signature motif, but all five Plasmodium perforins have a substitution to I, L or V at this position. An NCBI CD search retrieved no conserved domain or architecture when searched with the C-terminal domain aa 387–682. The alignment was performed using ClustalW (http://www.ebi.ac.uk/Tools/msa/clustalw2/) and visualization using Boxshade (http://www.ch.embnet.org/software/BOX_form.html). (TIF) [file pone.0201651.s001.tif]

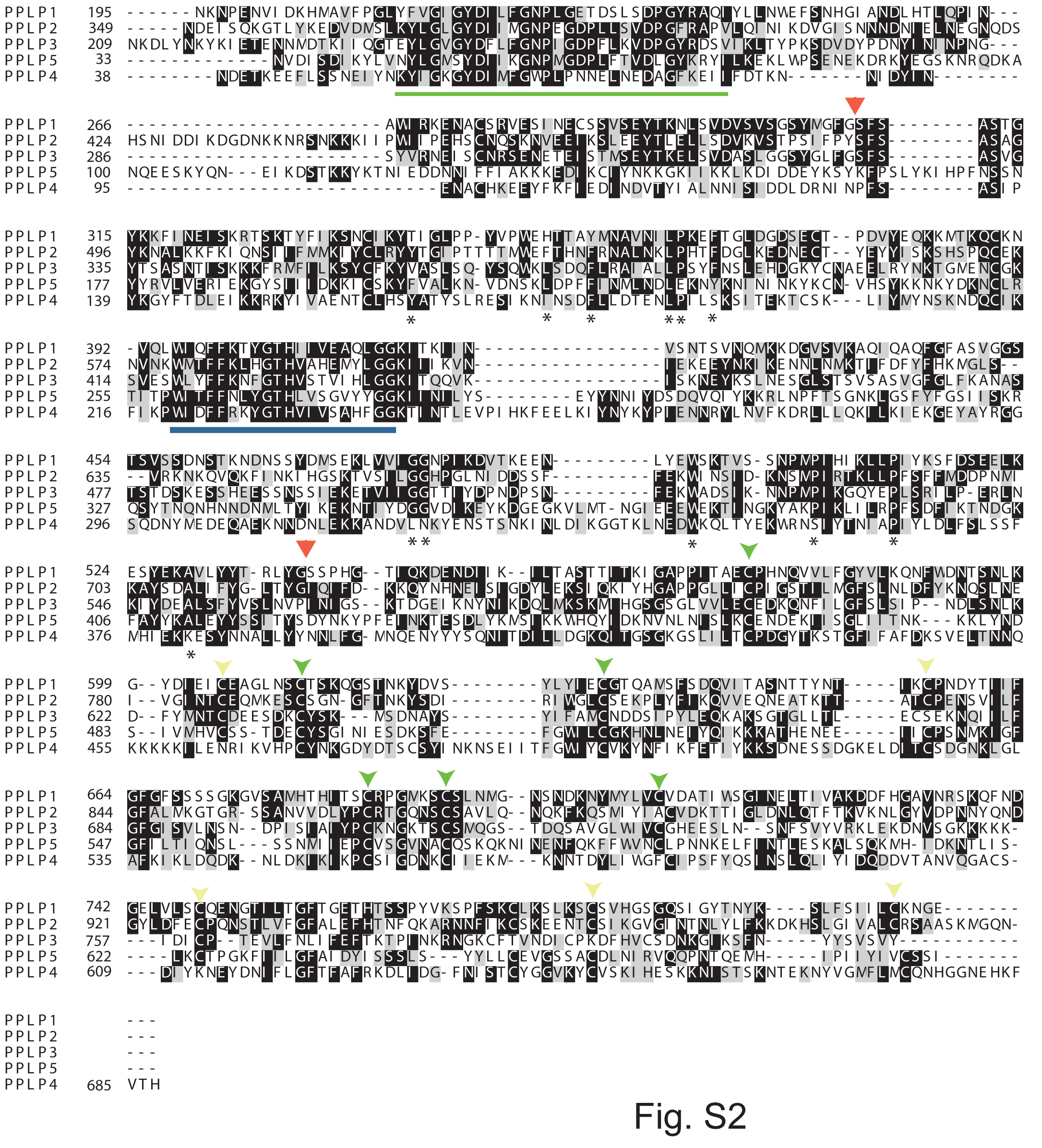

Supplement: S2 Fig — The N-termini were not included as indicated. PlasmoDB accession numbers and included aa region: PPLP1, PBANKA_1006300, aa 195–850; PPLP2, PBANKA_1432400 aa 349–999; PPLP3, PBANKA_0824200, aa 209 to 815; PPLP4, PBANKA_0711400, aa 33–682; PPLP5, PBANKA_0711600, aa 38 to 687. Green line, block of highly conserved region of unknown function; blue line, MACPF signature; orange arrowheads delineates the MACPF domain (Pfam01823); asterisks, conserved aa which have been recognized to be important for MACPF structure [1]; green arrowhead, conserved Cys residues, yellow arrowheads Cys residues conserved in four of the aligned sequences. Lines and asterisks are positioned below the alignment, arrowheads above. Alignment was performed as in S1 Fig. (TIF) [file pone.0201651.s002.tif]

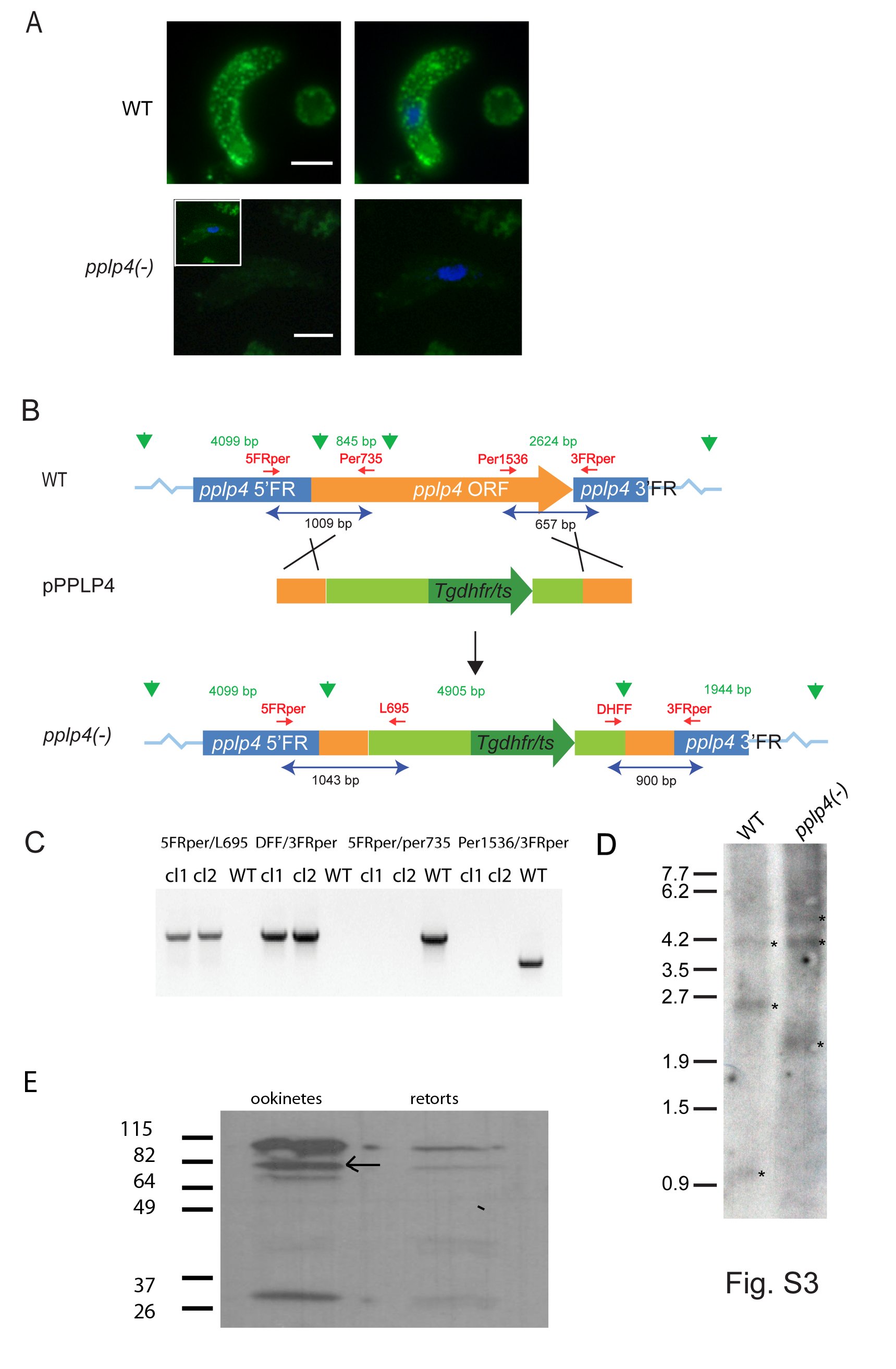

Supplement: S3 Fig — A. Immunolabeling of WT (top) and pplp4(-) (bottom) ookinetes using the PPLP4 antiserum. The WT displays the dotted label typical of PPLP4 while no signal was detected in the mutant. The two pictures were obtained from experiments conducted in parallel and pictures were taken with the same settings. Inset: the picture of pplp4(-) enhanced to show the ookinete. Scale bar, 5 μm. B. Schematic representation of the WT genomic locus (WT), the fragment introduced to target the gene (pPPLP4) and the locus after integration resulting in the disruption of the ORF (pplp4(-)). Primers and the sizes of fragments obtained are indicated in red, EcoRI sites as green arrowheads and restriction length fragments in green font. C. Genotyping using the primers depicted in A for PCR of gDNA from WT and pplp4(-) The sequence of the primers is available in S1 Table. D. Southern blot of genomic DNA of pplp4(-) and WT gDNA digested with EcoRI. The probe corresponds to 461 bp of the 5’-FR and the complete ORF. Asterisks indicate positive bands. E. Western blot analysis of P. berghei pplp4::mCherry 8h retorts and ookinetes extracts. The two samples were derived from the same culture and they were processed in parallel (see Materials and Methods). The blot was probed with an antibody recognizing mCherry. A processed form of the chimeric protein is also detected in both samples (arrow). Molecular weights are indicated on the left. (TIF) [file pone.0201651.s003.tif]

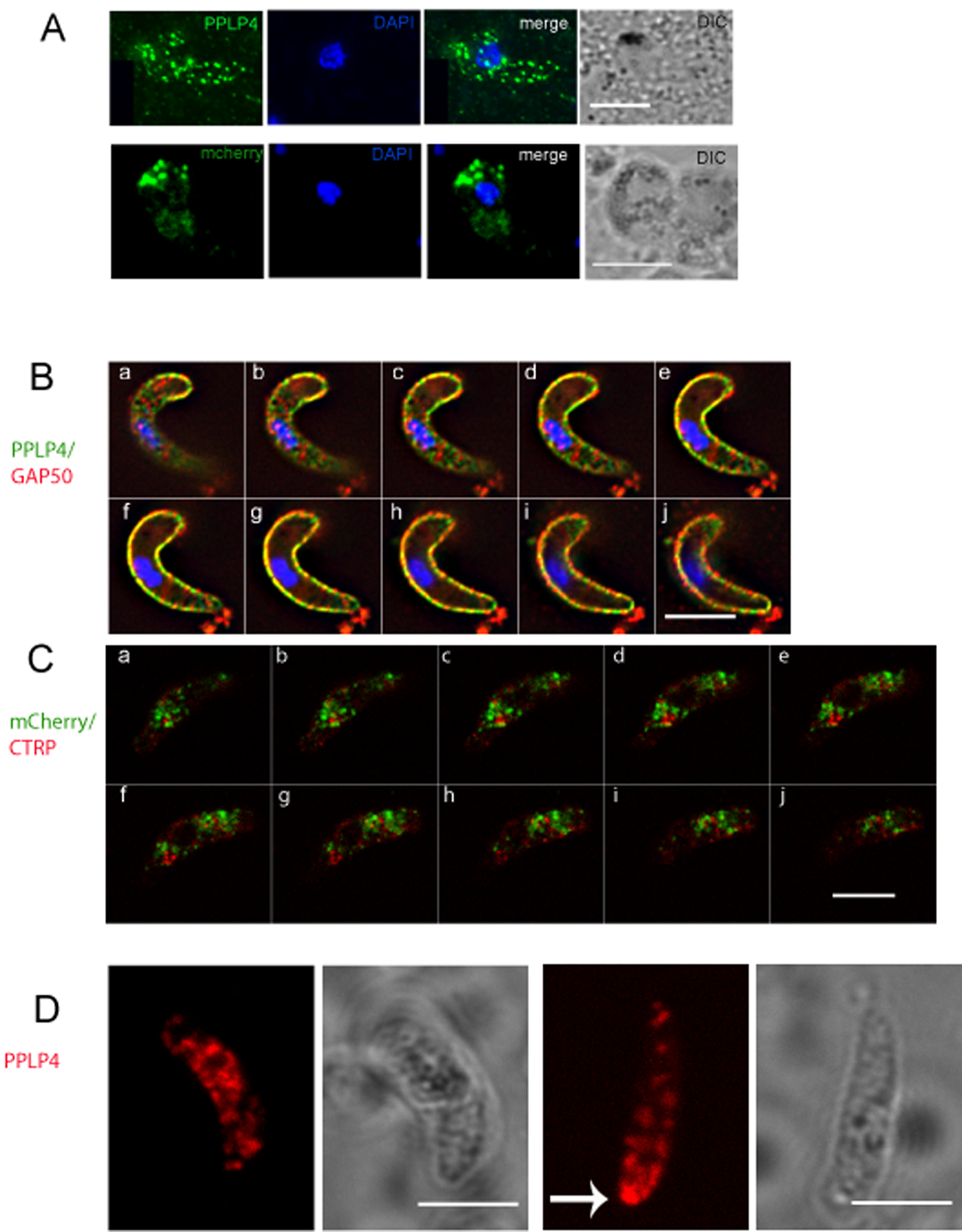

Supplement: S4 Fig — A Immunofluorescence analysis of P. berghei retorts labeled with the antiserum against PPLP4 (green) (top row). Bottom row shows a P. berghei pplp4::mCherry retort labelled with mCherry antibody (green). DNA was labeled with DAPI (blue). Scale bar 5 μm. Images show detection of PPLP4 as early as 8h retort stage. B. Montage of single sections of the same WT ookinete shown in Fig 1C, top row, labeled with antibodies against PPLP4 and GAP50. C. Montage of single sections of the same pplp4::mCherry ookinete shown in Fig 1C, bottom row, labeled with mCherry and CTRP antibodies. Nuclei stained with DAPI (blue). Scale bar 5 μm. D. Live images of pplp4::mCherry ookinetes. Variations in protein localization between different cells were observed. In some ookinetes, PPLP4::mCherry is observed as dispersed punctuate structures in the cytoplasm while in others it is also observed in the apical end (arrow). (TIF) [file pone.0201651.s004.tif]

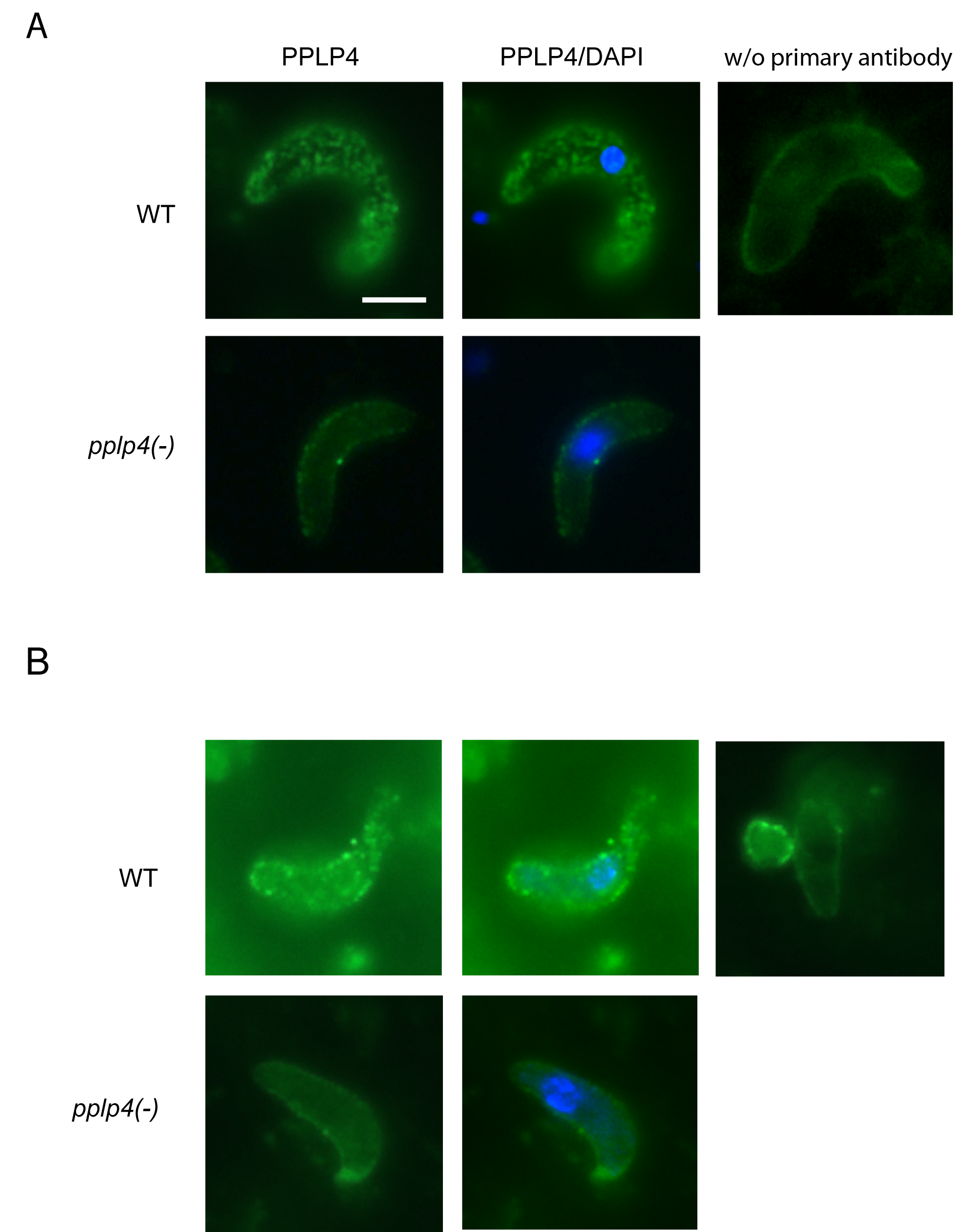

Supplement: S5 Fig — Top row, WT; bottom row, pplp4(-). B. Midgut ookinetes labeled using the same protocol as Wirth et al. Top row, WT; bottom row, pplp4(-). The nucleus is highlighted in blue. Scale bars, 5 μm. (TIF) [file pone.0201651.s005.tif]

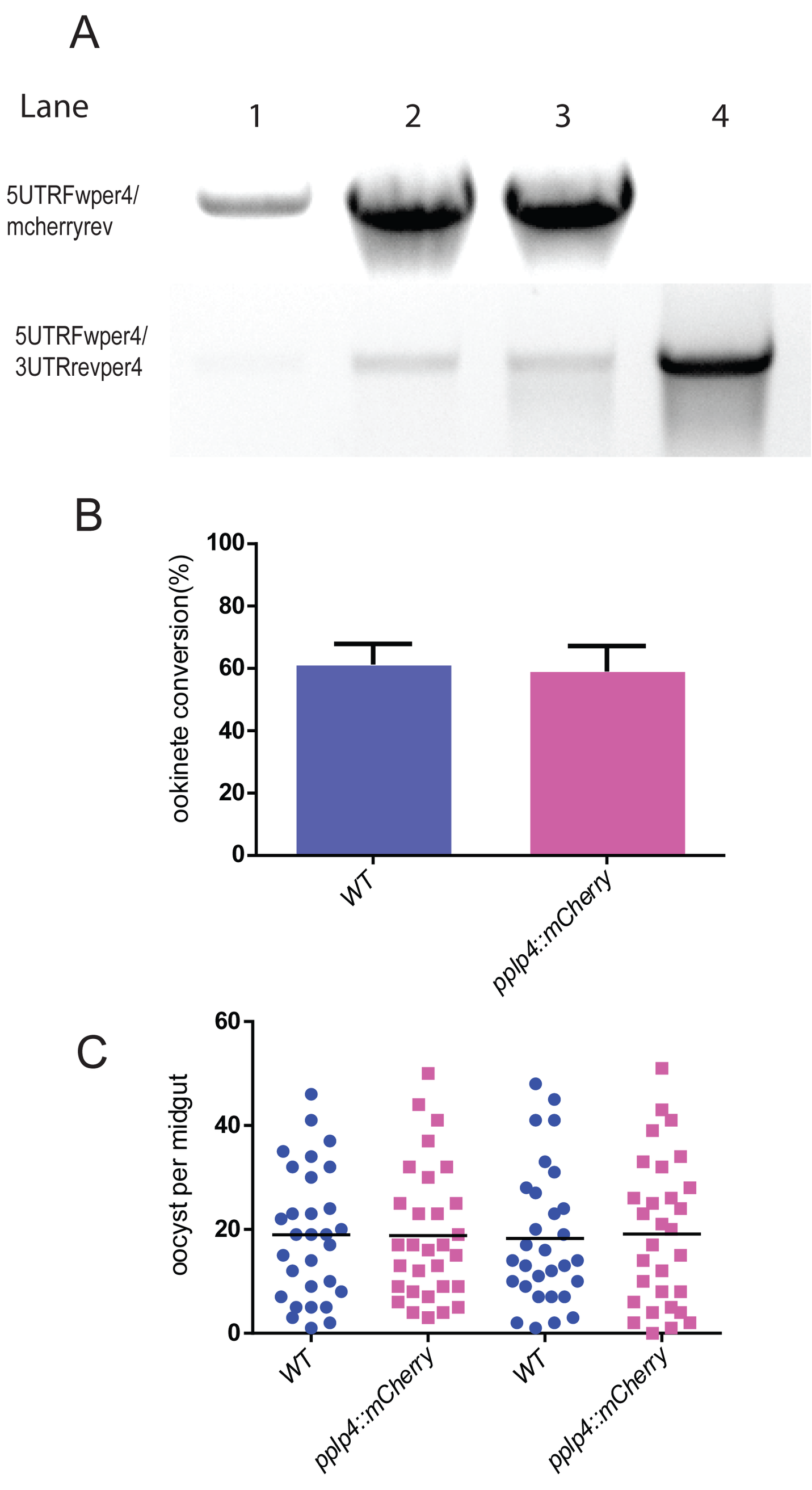

Supplement: S6 Fig — A. Genotyping with primers 5UTRFwper4/mcherryrev confirmed the presence of pplp4::mCherry parasites in oocysts (lane 1) and mutant sporozoites were able to infect a naïve mouse (lane 2). As controls genomic DNA from the transfectant pplp4::mCherry population and WT parasite (lanes 3 and 4 respectively) was used (top row). The quality of genomic DNA and the presence of WT parasites were tested using the primer pair 5UTRFwper4/3UTRrevper4 (bottom row). B. Ookinete conversion of pplp4::mCherry parasites similar to WT parasites. Values are average of three independent experiments. C. Oocyst formation of WT and pplp4::mCherry parasites was similar. Two independent experiments were carried out. (TIF) [file pone.0201651.s006.tif]

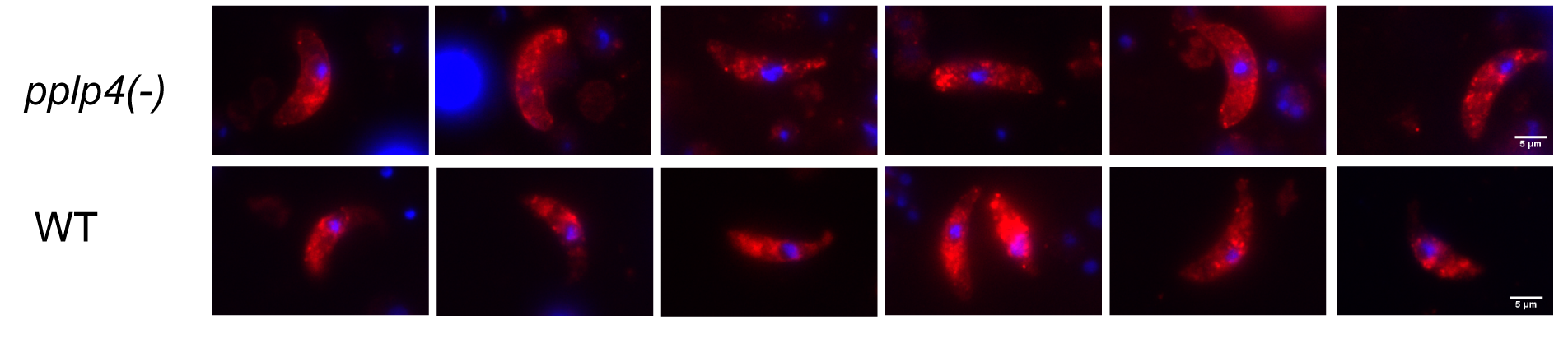

Supplement: S7 Fig — Ookinetes were labeled with the antibody directed against SOAP. Images were obtained in an epifluorescence microscope without deconvolution. 6 pplp4(-) mutant (top row) and 7 WT (bottom row) ookinetes are shown to illustrate the individual differences in SOAP localization in ookinetes. Scale bar, 5 μm. (TIF) [file pone.0201651.s007.tif]

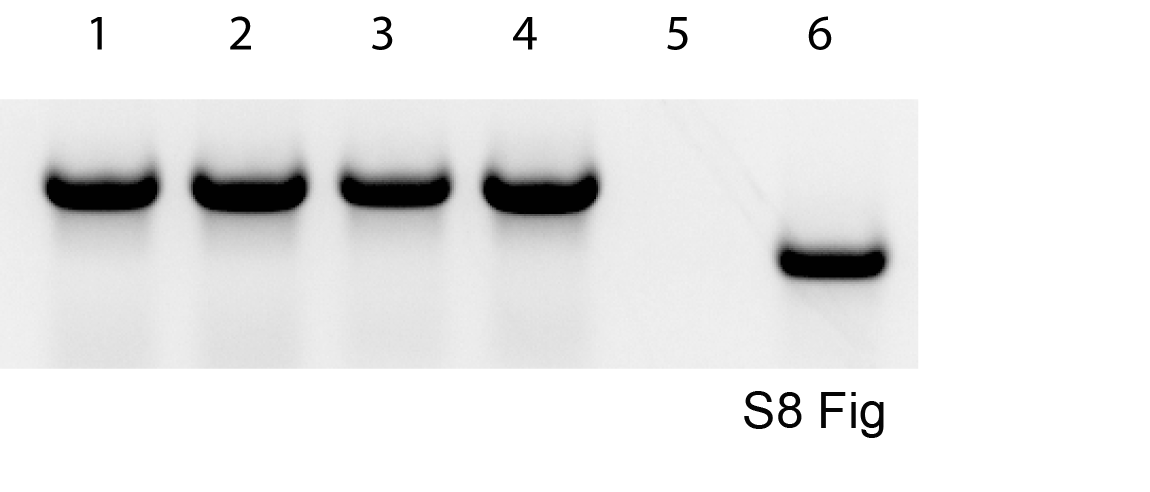

Supplement: S8 Fig — The mosquitoes had been infected by injection of ookinetes into the mosquito thorax. Lanes 1, 3 and 5 genomic DNA from pplp4(-) cl 17 bite-back mouse. Lanes 2 and 4, gDNA from pplp4(-) cl 17 Lane 6. WT gDNA. Primers in Lane 1 and 2 were 5FRper4/ L695testing for left integration of the gene replacement, in Lane 3 and 4 DHFF/3Rper4 (right integration). WT contamination was tested using primers Per4fw1536/3Rper4. (TIF) [file pone.0201651.s008.tif]
