# Supplementary material for: Essential role of Plasmodium perforin-like protein 4 in ookinete midgut passage
Source: PLoS One. 2018 Aug 13;13(8):e0201651. doi: 10.1371/journal.pone.0201651 (PMC6089593; doi:10.1371/journal.pone.0201651)
Supplement: S1 Table — (DOCX) [file pone.0201651.s012.docx]

**S1 Table.** Primers used in the construction and genotyping of the *pplp4(-)* disruption mutant and the *pplp4::mCherry* mutant as well as the RT-PCR detection of *pplp3* and *pplp5* transcripts.

| Primer name | Primer sequence | reference |
| --- | --- | --- |
| ***For the genotyping of the knock-out construct*** | | |
| Per4Fw1 | 5’gc**GGTACC**TATCTTATTTTGTGTTTCTTTATGGTT 3’ | This study |
| Per4Rev1 | 5’acgc**AAGCTT**ATGTAAGCATGTATTCTCTGCA 3’ | This study |
| Per4Fw2 | 5’acgc**GAATTC**ACCATGTTCTATAGGTGATA 3’ | This study |
| Per4Rev2 | 5’ac**GGATCC**ATTTCCTCCATGATTTTGACACA 3’ | This study |
| ***For the genotyping of the knock-out mutants*** | | |
| 5FRper4 | 5’ TAGCTATATTTTAGTCAATGTTTACC 3’ | This study |
| Per4rev735 | 5’ ATGTATTGGTACTTCTAACG 3’ | This study |
| L695 | 5’AATATTCATAACACACTTTTAAGC 3’ | Franke-Fayard *et al*., 2004 |
| DHFF | CCATCGATGTTTTTCTTACTTATATTTATAC | This study |
| 3Rper4 | 5’GTATTTATCTATTTATCTCATTCCACAA ‘3 | This study |
| Per4fw1536 | 5’TGATGGGAAAGAATTAGATA 3’ | This study |
| ***For the RT-PCR analysis of* pplp3** | | |
| per3aF | 5’ ACATGATGGCAAATATTGTAATGC 3’ | This study |
| per3aR | 5’ AAGGCTCGTATTGTCCTTTTATTG 3’ | This study |
| ***For the RT-PCR analysis of* pplp5** | | |
| per5F | 5’ AGGGTGTTAGTTGAACGAATTGA 3' | This study |
| per5R | 5' TCGATCCAAAATAAAAACTTCCA 3' | This study |
| ***For the genotyping of* pplp4::mcherry *mutant*** | | |
| mcherryR | 5’ CCCTCCATGTGAACCTTGAAG 3’ | Haussig *et al*., 2011 |
